# Application of weighted co-expression network analysis and machine learning to identify the pathological mechanism of Alzheimer's disease

**DOI:** 10.3389/fnagi.2022.837770

**Published:** 2022-07-13

**Authors:** Keping Chai, Xiaolin Zhang, Shufang Chen, Huaqian Gu, Huitao Tang, Panlong Cao, Gangqiang Wang, Weiping Ye, Feng Wan, Jiawei Liang, Daojiang Shen

**Affiliations:** ^1^Department of Pediatrics, Zhejiang Hospital, Hangzhou, China; ^2^Department of Neurological Surgery, Tongji Hospital, Tongji Medical College, Huazhong University Science and Technology, Wuhan, China; ^3^College of Life Science and Technology, Huazhong University of Science and Technology, Wuhan, China

**Keywords:** Braak stages, random forest, WGCNA, ssGSEA, neurodegeneration

## Abstract

Aberrant deposits of neurofibrillary tangles (NFT), the main characteristic of Alzheimer's disease (AD), are highly related to cognitive impairment. However, the pathological mechanism of NFT formation is still unclear. This study explored differences in gene expression patterns in multiple brain regions [entorhinal, temporal, and frontal cortex (EC, TC, FC)] with distinct Braak stages (0- VI), and identified the hub genes *via* weighted gene co-expression network analysis (WGCNA) and machine learning. For WGCNA, consensus modules were detected and correlated with the single sample gene set enrichment analysis (ssGSEA) scores. Overlapping the differentially expressed genes (DEGs, Braak stages 0 vs. I-VI) with that in the interest module, metascape analysis, and Random Forest were conducted to explore the function of overlapping genes and obtain the most significant genes. We found that the three brain regions have high similarities in the gene expression pattern and that oxidative damage plays a vital role in NFT formation *via* machine learning. Through further filtering of genes from interested modules by Random Forest, we screened out key genes, such as LYN, LAPTM5, and IFI30. These key genes, including LYN, LAPTM5, and ARHGDIB, may play an important role in the development of AD through the inflammatory response pathway mediated by microglia.

## Introduction

Via the distribution of neurofibrillary tangles (NFT) in the brain, Braak stages can not only be used for the pathological classification of Alzheimer's disease (AD) (Dickson, 1997), they are also related to memory and intellectual performance. However, to date, the pathological mechanism of NFT formation is still unclear (Duyckaerts et al., 1997; Grober et al., 1999). A large body of evidence indicates that at different stages of AD, the distribution region of NFT in the brain is also different. For example, the entorhinal cortex (EC) is the area where NFT deposits occur first in AD (Braak and Braak, 1991). However, the pathological mechanism of its formation is still unclear. Several hypotheses, such as oxidative damage, oxidative stress, insulin resistance, apoE, neuroinflammation, and other theories were established (Solomon et al., 2014; Nakamura et al., 2018). Exploring the gene expression patterns of different brain regions, especially EC, may better help understand the mechanism of NFT formation.

Weighted gene co-expression network analysis (WGCNA) is a biology algorithm used to describe the correlation between clinical characters and gene expression based on the microarray data (Langfelder and Horvath, 2008). WGCNA can be used for clustering genes with highly correlated expression, for relating the modules to phenotypes to get the most phenotypic trait-related module, and for summarizing these co-expressed gene clusters by identification of the module eigengene or hub genes. Random forest (RF) is a more advanced machine learning algorithm based on a decision tree (Sarica et al., 2017). Like other decision trees, random forests can be used for both regression and classification.

In this study, we performed ssGSEA, machine learning, and WGCNA analysis on publicly accessible transcriptome data obtained from the human different cortex regions of individuals at different Braak stages. We found the similarities and differences in the transcription patterns of the genome in the three different brain regions [EC, temporal and frontal cortex (TC, FC)] in Braak stages 0-VI. By evaluating the ssGSEA results of EC, we found that the oxidative damage pathway plays a vital role in classifying the Braak stages *via* the random forest and best subset algorithm, the imp is 0.57. Through calculating the correlation coefficients between the modules and the oxidative damage pathway, we obtained a module of interest. We then disclosed the overlapping genes between differentially expressed genes (DEG, between Braak stage 0 and Braak stage I–VI) and genes of interest in the module. Using these overlapping genes, we conducted metascape analysis and further identified the central players within the module through network analysis. Our findings reveal that *C1QA, C1QB, LYN, CD68, LAPTM5, IFI30, PI3KAP1, HCK*, and *ARHGDIB* are significantly associated with oxidative damage and immune response, which may be novel biomarkers involved in AD.

## Results

### Identification of consensus modules across different cortical regions

**Figure 1:**
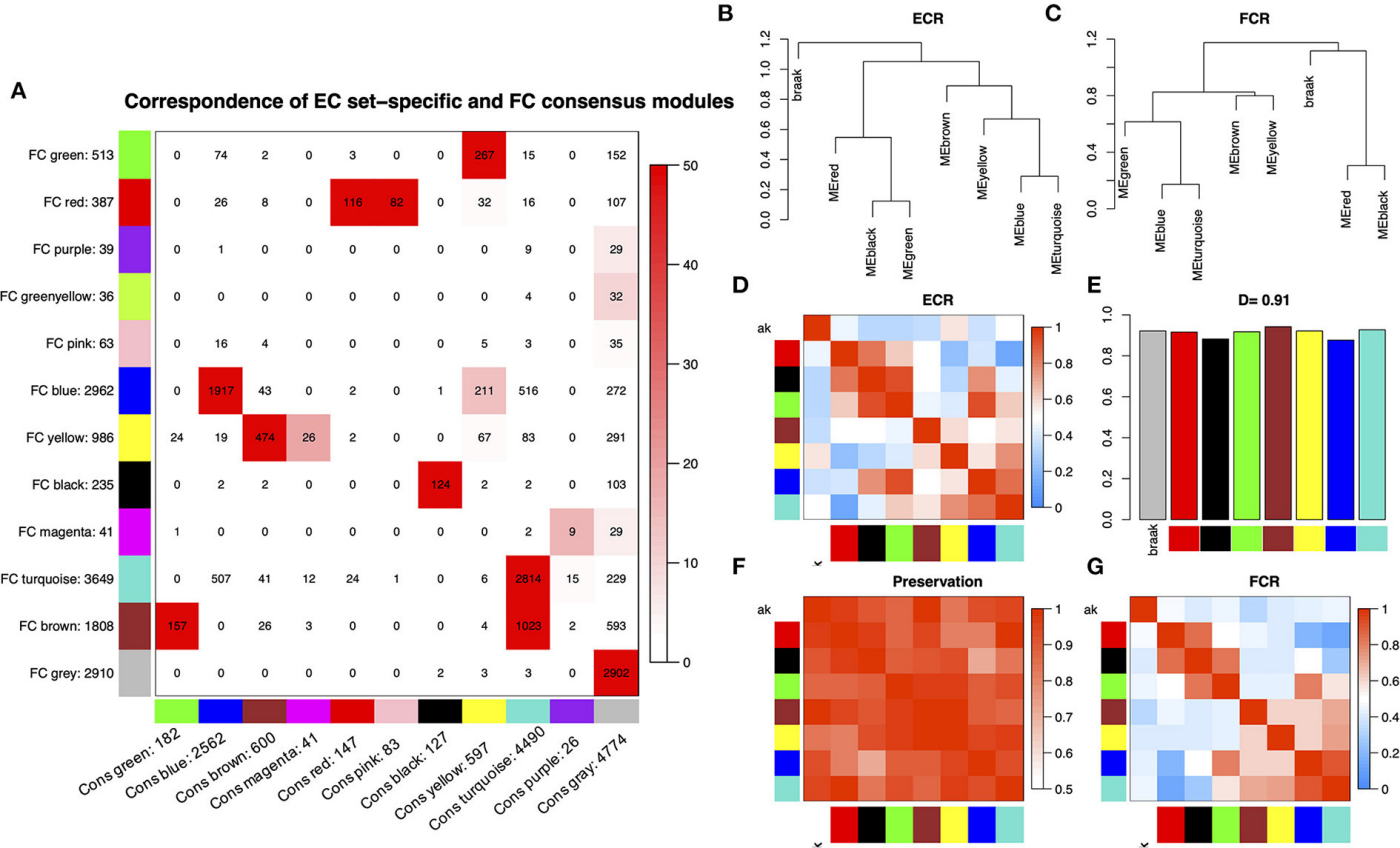
**(A)** Comparison between EC set-specific modules and EC-FC consensus modules of the global co-expression network. The numbers in the table represent genes that are shared between EC modules and consensus modules. The color code of the table is -log(p), where p is the *p*-value of Fisher's exact test of the overlap of the two modules. The darker the red, the more pronounced the overlap. **(B,C)** Clustering dendrograms of consensus module eigengenes for identifying meta-modules show the presence of similar major branching patterns in the EC and FC eigengene network. **(D,G)** The heatmap shows the eigengene adjacencies in EC and FC eigengene networks. Each row and column correspond to an eigengene tagged by consensus module color. Within each heatmap, red represents high adjacency (positive correlation) and blue represents low adjacency (negative correlation) as represented by the color legend. **(E)** Bar plot shows the preservation degree of each consensus eigengene as the height of the bar (y-axis) where each colored bar corresponds to the eigengene of the associated consensus module. The high-density value *D* (Preserve EC, FC) = 0.91 indicates the high overall preservation between the EC and FC networks. **(F)** Adjacency heatmap of the preservation network between EC and FC consensus eigengene networks. The saturation of the red color indicates a correlation preservation of EC and FC module eigengenes.

Before WGCNA, the genes detected in GSE131617 were filtered according to the filtering procedure described in Method, and 13,629 genes were obtained. Then the microarray data of 46 samples in each cortical region were read by R for Hierarchical clustering (Supplementary Figure S1a). The consensus network of scale independence and mean connectivity analysis showed that when the weighted value equals to 14, the average degree of connectivity was close to 0, and scale independence was greater than 0.9, so the weighted value was set to 14 (Supplementary Figure S1b). WGCNA was performed to identify consensus modules. A comparison between EC set-specific modules and EC-FC consensus modules of the global co-expression network indicated that most EC modules were preserved in FC (Figure 1A). The strong overlap of the corresponding gene modules showed the similarity of cluster patterns in the EC and FC regions. Figures 1B–G and Supplementary Figures S1c, S2, S3 show that the overall preservation of the three networks is a positive correlation. The mean density of the three networks exceeded 0.9 in all 3 cortical regions, demonstrating that the overall structures of the co-expression networks were similar for the three cortical regions. These results indicated that the differences in these cortical regions may exist in the particular genes within the consensus network.

### ssGSEA functional enrichment analyses and key pathway identification and validation help to find the module of interest verified in WGCNA analysis in EC

In the above results, we found that the overall structures of the co-expression networks were similar for the three cortical regions. In addition, an abundance of studies have shown that in the Braak stages I–II, aberrant deposits of NFT first appear in the entorhinal cortex, which is significant for finding the potential biomarkers and therapeutic targets of AD.

**Figure 2:**
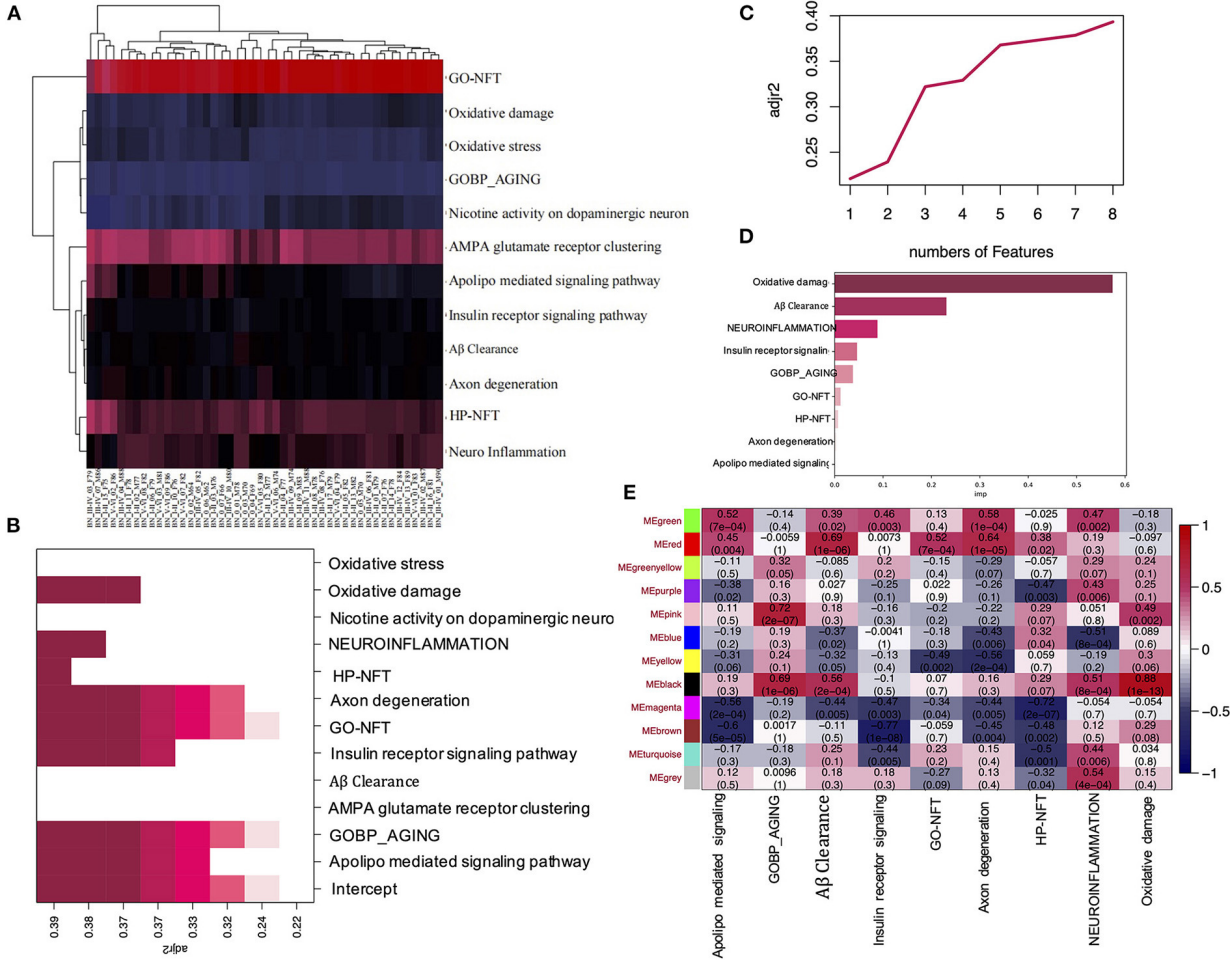
ssGSEA and WGCNA analysis of the EC data. **(A)** Heatmap shows the ssGSEA scores of the different gene sets in corresponding samples. **(B)** The heatmap shows the adj R2 of the Best subset regression result of each ssGSEA pathway. **(C)** The plot shows the adj R2 of the number of features in the Best subset regression model. **(D)** The bar plot shows the importance of each ssGSEA pathway by the RF model. **(E)** Pearson correlation coefficient between the pathway and module eigengenes, numbers in brackets indicate the corresponding *p-*values.

To explore the signaling pathways most related to Alzheimer's disease, first, the ssGSEA analysis was performed (Figure 2A). The gene set of pathways related to Alzheimer's disease can be seen in Supplementary Table S1. The second, best subset regression was conducted to identify the representative subset (Figures 2B,C). From the results, we can see that the feature number of best subsets is 8, and GO-NFT, HP-NFT, oxidative damage, and axon degeneration pathway are saved in the best subset. Next, we performed the random forest algorithm based on the sklearn and boruta packages to analyze the best subset of data to find the most important features, as shown in Figure 2D and Supplementary Table S2, the oxidative damage pathway was found to be the most important feature.

To identify the modules which are most significantly associated with the oxidative damage pathway in EC, the Pearson's correlation coefficient between the module and oxidative damage was calculated. The highest positive association in the module trait relationship was found between the black module and oxidative damage score (cor = 0.88, *p* < 0.001, Figure 2E), and we also found that the black module had a high correlation with the aging and *Aβ* clearance pathway (cor = 0.69, 0.56, *p* < 0.001, Figure 2E). Thus, the black module was selected as a module of interest in subsequent analysis.

### Identifying hub genes in the black module

**Figure 3:**
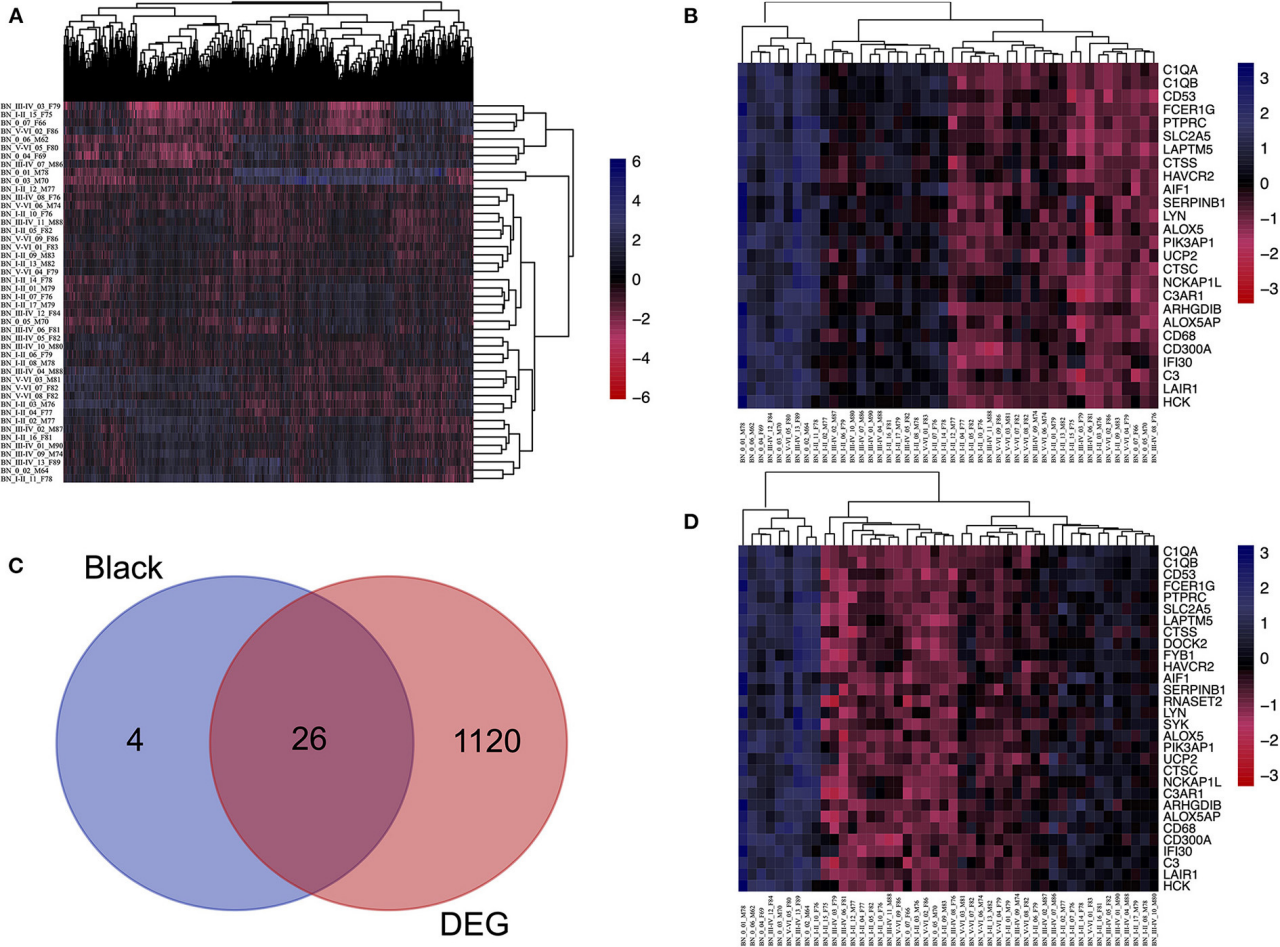
Identifying the overlapping genes between downregulated DEGs in the aged group and genes in the black module. **(A)** Heatmap of the expression of DEGs. **(B)** Heatmap of the Top30 gene expression in the black module. **(C)** Using veen tools to find the overlap genes between downregulated genes in DEGs and genes in the black module. **(D)** Heatmap showing the expression of the overlapping genes in different samples.

First, to find the DEG between Braak stage 0 and Braak stages I–VI, the EC samples were grouped into individuals at Braak stage 0 and Braak stages I–VI, and Limma packages were performed. About 10% of the genes were significantly changed (*p* < 0.05, Figure 3A), and the 201 DEGs were enriched in interleukin-4 related pathways (Supplementary Figure S4). We then performed overlap analysis between DEGs and Top30 genes in the black module by online veen tool, we found 26 genes that were in DEGs and also in the black module (Figures 3B–D). These genes are highly related to oxidative damage, suggesting that they might play an important role in oxidative damage AD. We found that 21 genes of DEGs in the data set GSE53480 (expression data from Tg4510 transgenic mice) also exist in the DEGs of GSE131617 (Supplementary Figure S5). The Tg4510 mouse is a classical model which is used to express pathological tau in neurons, having a high correlation with NF formation. Therefore, we chose this model to support our findings. To prove that there is no gender bias among the 26 genes of interest, we compared the DEGs between the genders, and 36 genes that were co-expressed in male and female patients were identified. Among the 36 genes, 22 genes were also found in the 26 interested genes (Supplementary Figure S6 and Supplementary Table S5).

### Identifying the hub gene functional annotation

**Figure 4:**
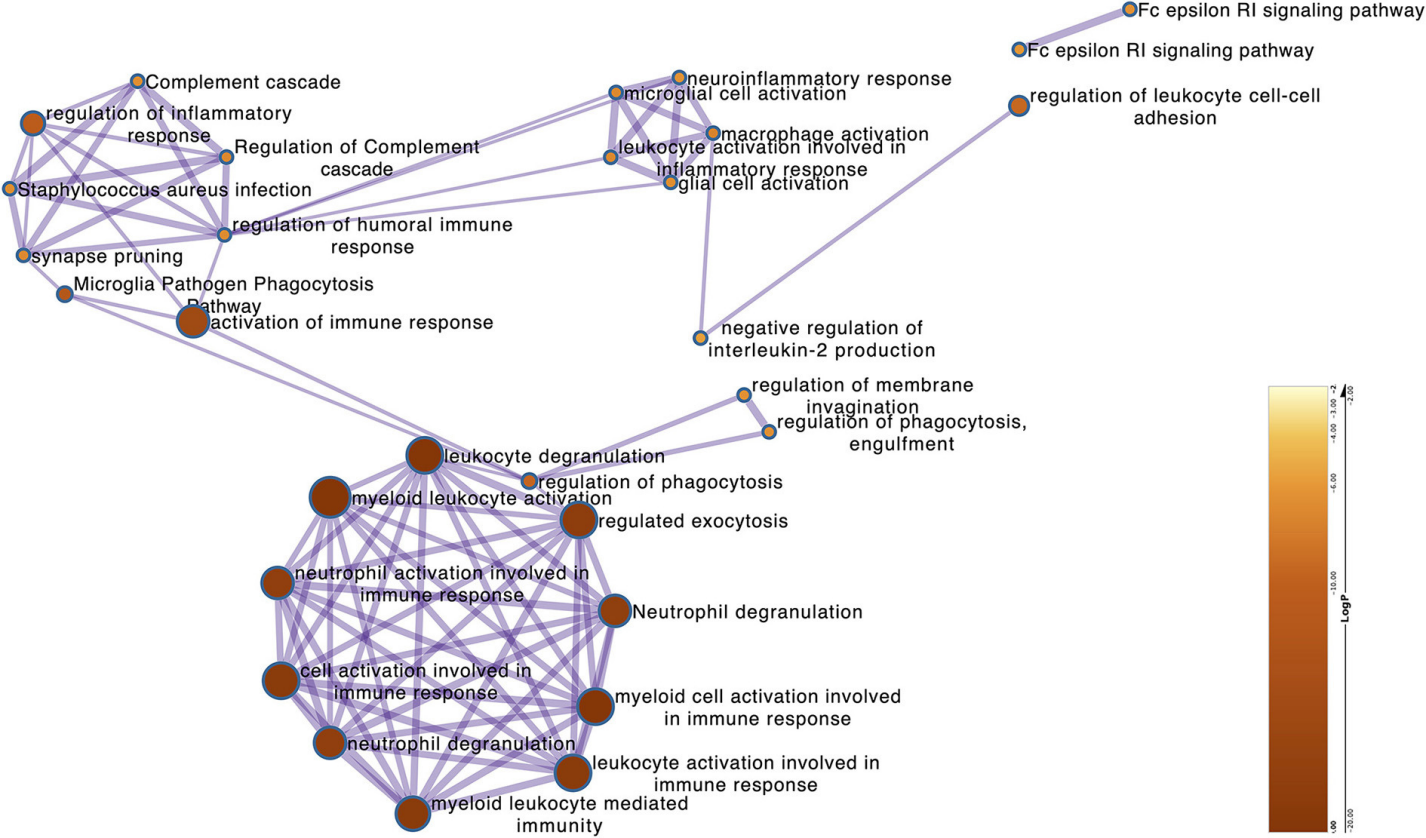
The Metascape of the overlapping genes. The network shows the GO terms that the log *P* (−23 to −8) correlates with the significance of the enrichment.

The above-identified overlapping genes were subjected to GO functional and KEGG pathway enrichment analysis. The biological processes of overlapping genes were found to focus on the regulation of inflammatory response and leukocyte degranulation. The molecular functions of overlapping genes were found to focus on IgE binding, non-membrane spanning protein tyrosine kinase activity, and phosphotransferase activity (Figure 4 and Supplementary Figure S7).

### Identification of the most significant genes and network construction

**Figure 5:**
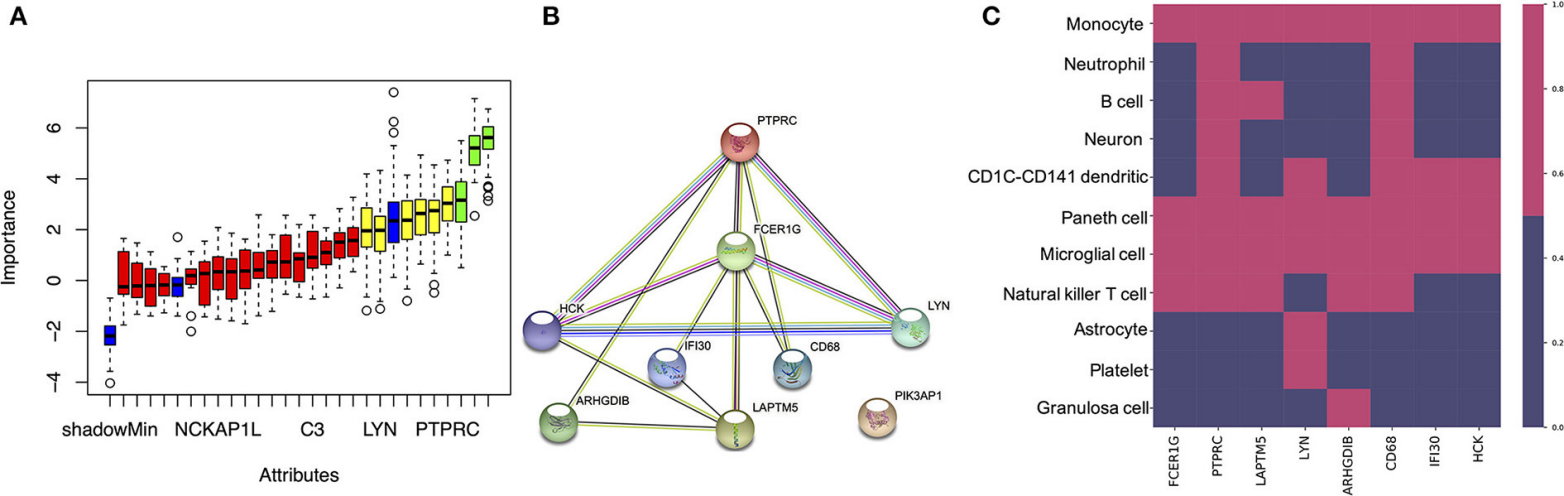
Identifying the most important genes *via* RF and the cellular distribution of the important genes in the brain. **(A)** Random Forest algorithm result. The blue box plot corresponds to the minimum, average, and maximum Z scores of a color attribute. The red, yellow, and green boxes represent the Z scores of rejected, tentative, and confirmed genes, respectively. **(B)** The PPI network of important genes *via* String. **(C)** The heatmap shows the distribution of the selected genes in different cell types.

To identify the most important genes related to oxidative damage, the overlapping genes were further filtered by RF classification. Gene counts were input into the RF classifier model, and the unimportant genes, such as *C1QA, C1QB, CTSC, SLC2A5, UCP2*, and others, were removed (Figure 5A and Supplementary Table S3). To ascertain the significance of genes and analyze the network in the corresponding modules, the PPI maps were constructed *via* String (Figure 5B). Hub genes in the network, including *PTPRC, LYN, LAPYM5, HCK, IFI30, ARHGDIB*, and *PIK3AP1* were constructed. In the cell marker database, we found that the distribution of the above genes in brain cells was very similar, mainly in microglia cells (Figure 5C).

## Discussion

NFT is the major pathological characteristic of neurodegenerative diseases, such as PD (Parkinson's disease)/AD (Wang and Mandelkow, 2016). Exploring the mechanism of NFT formation is extremely important for discovering the therapeutic targets in these diseases. In this study, we performed WGCNA, ssGSEA, and machine learning analysis on the dataset GSE131617, which includes 46 samples from individuals at Braak stages between 0 and I-VI. Data from multiple samples based on the different brain regions (EC, FC, TC) is a good candidate for WGCNA analysis. First, consensus modules between different brain regions were constructed, and 7 consensus modules were identified between EC and FC. Figures 1B-G and Supplementary Figures S1c, S2, S3 showed that the overall preservation of the three networks was a positive correlation. The mean density of the three networks exceeded 0.9 in all 3 consensus modules, demonstrating that the overall structures of the co-expression networks were similar for the 3 cortical regions. However, the purple, pink, green-yellow, and magenta module of EC were not recognized in the consensus module (EC, FC), indicating that the difference between the two regions was related to these modules. Furthermore, the black and red modules in EC that are most related to oxidative damage and the *Aβ* clearance pathway have not been recognized in the consensus module identified by EC and TC (Figure 2E and Supplementary Figure S2). These showed that TC was quite different from EC in the signal pathway of oxidative damage and *Aβ* clearance.

A number of studies show that NFT formed by the aggregation of tau is the main pathological character of AD, the peak of tau aggregation occurs in the Braak stages I of individuals in their 40–50 s, as opposed to in later life (Wischik et al., 2014). Furthermore, many studies have shown that the EC is the region where NFT deposits occur first during the process of neurodegeneration (Cui et al., 2015). Therefore, studying the differences in gene transcription levels between Braak stages I and Braak Stages 0 in the EC is extremely important to reveal the pathogenesis and therapeutic targets of AD. It should be added that we use the Braak stage as a simple qualitative marker of AD to identify the DEGs between the Braak stage 0 and Braak stage I–VI. In this study, when we performed ssGSEA and random forest analysis on the dataset of EC samples, we found that the unexpected oxidative damage signaling pathway was most important when distinguishing between Braak stage 0 and Braak stages I- VI rather than the signaling pathway related to NFT (Figures 2A–D). This indicates that among the important basis of Braak stages, the formation of NFT is more likely due to changes in the expression level of genes related to the oxidative stress pathway, rather than the NFT signaling pathway. When we analyzed the overlapping genes in the black module which were most related to oxidative damage and the DEG, we found that these genes were not only related to oxidative damage but also related to immune response and microglia-mediated inflammation (Figures 2E, 3, 4). To identify the genes that were most intensively related to Braak stages, we further used one of the machine learning algorithms, Random Forest, and inputted the expression matrix of the overlapping 26 genes as features into the model for training, and finally screened out 9 key genes (Figure 5A and Supplementary Table S3). When analyzing these 9 molecules, we found that most of them are expressed in microglia (Figure 5C), which further indicated that microglia might play an important role in the Braak stages (0 vs. I–VI).

It has been reported that activated microglia can induce the formation of NFT (Fan et al., 2017), and several hypotheses can explain how the activated microglia mediates the formation of NFT, such as complement pathway, IL-CDK5 pathway, and exosome secretion, etc. (Quintanilla et al., 2004; Asai et al., 2015; Saha and Sen, 2019; Vogels et al., 2019). However, this requires further research, examining how molecules such as *LYN, HCK*, and *PTPRC*, which are distributed in the microglia, promote the formation of NFT. LYN and HCK, as Non-receptor tyrosine-protein kinases, can combine with NLRP3, which is involved in the phosphorylation of tau and the formation of NFT to promote the release of IL1B from microglia (Fitzer-Attas et al., 2000; Jevtic et al., 2017; Gwon et al., 2019; Kong et al., 2020). In the co-expression network, *PTPRC* and *LAPTM5* were identified as hub genes. PTPRC is not only an important regulator of T cell and B cell antigen receptor signal transduction but also an enzyme that dephosphorylates LYN. It has been reported that LAPTM5 can not only regulate the production of pro-inflammatory cytokines in macrophages but also regulate the antigen receptor signal transduction of T cells and B cells (Zouali, 2014). There is a lot of data showing that *LAPTM5* and *PTPRC* are not only co-expressed in AD/PD (Figure 5B), but also in systemic lupus erythematosus, lung cancer, and other diseases (Salih et al., 2019; Zhang et al., 2020, 2021). This indicated that LAPTM5 and PTPRC may play a similar role in the phosphorylation of LYN. Moreover, in this study, we found that a decrease in the expression of these co-expressed genes at Braak stage I- VI, which was negatively correlated with the degree of NFT needs further discussion. It has been reported that the expression of *LYN* in activated microglia is less than that of homeostasis microglia (Sierksma et al., 2020). This indicated that LYN may play a role in activated microglia, and the decrease of *PTPRC* and *LAPTM5* may lead to an increase of phosphorylated LYN so that it can promote the release of inflammatory factors.

In this study, we also found that *IFI30 and FCERIG* in the co-expression network were also distributed in microglia (Figures 5B,C). It has been reported that both of them are highly expressed in microglia around *Aβ* (Satoh et al., 2018), which may imply that the two of them are involved in the function of *Aβ* clearance (Figure 2E). However, in this study, we found that their expression in Braak stage I–VI decreased. How their reduction in microglia promotes the formation of NFT requires further study.

To our surprise, *ARHGDIB* was found to be mainly co-expressed with *LAPTM5* and *PTPRC* in the co-expression network. Its related pathways are involved in the GPCR signaling pathway, apoptosis, and survival Caspase cascade (Kardol-Hoefnagel et al., 2020). Through network analysis (Figures 5B,C), we speculated that it may have similar functions to *LAPTM5* and *PTPRC*. A decrease in the expression of *ARHGDIB* may also play a role in the formation of NFT. Further studies are needed to reveal the function of *ARHGDIB* in microglia.

In conclusion, through WGCNA and machine learning analysis, we found that the EC, FC, and TC regions of Braak stages 0-VI had similar genome transcription patterns. Furthermore, we found that oxidative stress might play a key role in the development of AD, which may be mediated by *ARHGDIB, IFI30*, and *LAPTM5*, etc. through microglia.

## Materials and methods

### Data acquisition and preprocessing

The data used in this paper were obtained from the GEO database in NCBI (Gene Expression Omnibus, http://www.ncbi.nlm.gov/geo), and the data entry number is GSE131617 (Kikuchi et al., 2020, p. 1). The platform is Affymetrix Human Exon 1.0 ST Array [transcript (gene) version, HuEx-1_0-st]. Gene expression in the cortex of Braak stages 0, I–II, III–IV, and V–VI was detected. The normalized and log2-transformed data from 71 samples were downloaded and the expression matrix was obtained, and data filtering was performed before WGCNA analysis. For data filtering, first, 61 samples with a neuropathological diagnosis of minimal senile change and AD were performed. Second, the gene type of APOE was 3^*^3 and 15 samples were removed. Forty six samples in the dataset were kept and the clinical characteristics of these samples are shown in Supplementary Table S4. Probes without corresponding annotation information were removed. There were about 13,629 genes in the dataset.

### Single sample gene set enrichment analysis

ssGSEA is an implementation method proposed for a single sample GSEA (Subramanian et al., 2005; Barbie et al., 2009). The difference between GSEA and ssGSEA is that ssGSEA does not need to prepare an expression matrix file. The functions of the gene set were acquired from a Molecular Signatures Database (MSigDB) as described in the review, including aging, insulin receptor pathways, oxidative stress, oxidative damage, NFT, and Nicotine activity on dopaminergic neurons, etc. The performances of the pathway in the gene set were quantified by the ssGSEA algorithm (R package “gsva”) based on transcriptome profiling data and pathway gene sets.

### Application of best subset regression to find the best subset of the ssGSEA pathway

The entorhinal cortical samples were grouped into individuals of Braak stage 0 and Braak stages I–VI. We used the Braak stage as a binary category for simple AD diagnosis and classification. Inputting the ssGSEA scores into the best subset regression model *via* leaps package to predict which group the samples belong to, and the best number of features as the input for subsequent analysis.

### Application of random forest algorithm to find the most important pathway and genes related to braak stages

The entorhinal cortical samples were grouped into individuals of Braak stage 0 or individuals of Braak stages I- VI. Inputting the overlapping genes counts and ssGSEA enrich scores into the random forest classifier model *via* Boruta package to predict which group the samples belonged to and the most important overlapping genes and identify the ssGSEA pathway for the most accurate model for grouping.

### Construction of weighted gene co-expression network and identification of significant modules

Data were processed using R 3.4.2 software. To ensure that the results of network construction are reliable, abnormal samples were removed. Then, the weighted gene co-expression network was constructed by the WGCNA package based on R 3.4.2. First, the Pearson correlation coefficient was calculated to assess the similarity of the gene expression profiles. Second, the correlation coefficients between genes were weighted by a power function to obtain a scale-free network. A gene module is a cluster of densely interconnected genes in terms of co-expression. Then, the hierarchical cluster was used to identify gene modules and different modules were represented by different colors. The dynamic treecut method was used to identify different modules, the adjacency matrix was converted to a topology overlay matrix (TOM) and modules were detected by cluster analysis during module selection.

### Correlation analysis of gene modules with clinical phenotype

To detect the associations of modules and clinical phenotype (ssGSEA scores), first, the clinical phenotype data and gene expression data were correlated using the match function. Secondly, the associations of the module eigengene (ME) and the clinical phenotype were calculated by Pearson's correlation analysis. Modules showing significant association to oxidative damage pathway were obtained. At last, to further confirm the modules with significant correlation to oxidative damage, the correlation coefficient between the module membership (gene expression level) with gene significance (GS, for assessing the association of genes with phenotypes) was calculated using the labeled heatmaps function, and *p-*values were obtained.

### Finding the overlapping genes between the differentially expressed genes (DEG, between braak stage 0 and braak stages I–VI) and genes of interest in the module verified by WGCNA

The entorhinal cortical samples were grouped into individuals at Braak stages 0 and individuals at Braak stages I- VI and Limma packages were performed to find the DEG (Diboun et al., 2006; Ritchie et al., 2015). Samples of Braak stage 0 were regarded as control, 201 genes with a corrected *p*-value of less than 0.05 were found in samples of Braak stages I–VI. Next, the overlapping genes between downregulated DEG and genes of interest in the module were discovered by using online veen tools (http://bioinformatics.psb.ugent.be/webtools/Venn/).

### Metascape analyzes, identification of hub genes, and protein-protein interaction analysis

For the obtained overlapping genes, functional enrichment of Gene Ontology (GO) and KEGG pathways analyses were performed using Metascape (https://metascape.org) (Zhou et al., 2019). Log *P* between −23 and −8 were considered to be significant enrichment. These enrichment results were also analyzed using Cytoscape for the identification of important pathways (Warde-Farley et al., 2010). The identified hub genes were further confirmed and analyzed using a String network constructed by the online database String (http://string-db.org) (Szklarczyk et al., 2017).

### Exploring the cellular distribution of the identified genes

By using the Cell marker database (http://biocc.hrbmu.edu.cn/CellMarker/search.jsp), the cellular distribution of the identified important genes was further explored.

## Data availability statement

The datasets presented in this study can be found in online repositories. The names of the repository/repositories and accession number(s) can be found in the article/Supplementary Material.

## Author contributions

KC: conceptualization, methodology, investigation, data curation, visualization, and writing–original draft. XZ: conceptualization, investigation, and writing–original draft. HT and GW: software. HG: resources and software. WY: data curation. SC: supervision. FW, DS, and JL: supervision and writing–review and editing. All authors contributed to the article and approved the submitted version.

## Conflict of interest

The authors declare that the research was conducted in the absence of any commercial or financial relationships that could be construed as a potential conflict of interest.

## Publisher's note

All claims expressed in this article are solely those of the authors and do not necessarily represent those of their affiliated organizations, or those of the publisher, the editors and the reviewers. Any product that may be evaluated in this article, or claim that may be made by its manufacturer, is not guaranteed or endorsed by the publisher.
