# Supplementary material for: Application of weighted co-expression network analysis and machine learning to identify the pathological mechanism of Alzheimer's disease
Source: Front Aging Neurosci. 2022 Jul 13;14:837770. doi: 10.3389/fnagi.2022.837770 (PMC9326231; doi:10.3389/fnagi.2022.837770)
Supplement: Supplementary file 6 [file Data_Sheet_1.docx]

**FigureS1. EC-FC consensus modules construction. a)** Clustering of samples in the EC and FC expression data. Branches of the dendrogram, cut at the red line, correspond to outliers. **b)**. Numbers in the scatterplots represent for the power of soft thresholding. The scatterplots depict that when the soft threshold reaches around 14, both of the data sets can obtain scale-free network. **c)** Pearson correlation coefficient between the clinical traits and consensus module eigengenes, numbers in brackets indicate the corresponding *p* values.

**Figure.S2. TC-FC consensus modules construction. a)** Pearson correlation coefficient between the clinical traits and consensus module eigengenes, numbers in brackets indicate the corresponding *p* values. **b-c)** Clustering dendrograms of consensus module eigengenes for identifying meta-modules show presence of similar major branching pattern in TC and FC eigengene network. **d, g)** The heatmap shows the eigengene adjacencies in TC and FC eigengene networks. Each row and column correspond to an eigengene tagged by consensus module color. Within each heatmap, red represents high adjacency (positive correlation) and blue represents low adjacency (negative correlation) as represented by the color legend. **e)** Bar plot shows the preservation degree of each consensus eigengene as the height of the bar (y-axis) where each colored bar corresponds to the eigengene of the associated consensus module. The high-density value *D* (Preserve TC, FC) = 0.91 indicates the high overall preservation between the TC and FC networks. **f)** Adjacency heatmap of the preservation network between TC and FC consensus eigengene networks, the saturation of the red color indicates correlation preservation of TC and FC module eigengenes.

**Figure.S3. TC-EC consensus modules construction. a)** Pearson correlation coefficient between the clinical traits and consensus module eigengenes, numbers in brackets indicate the corresponding *p* values. **b, c)** Clustering dendrograms of consensus module eigengenes for identifying meta-modules show presence of similar major branching pattern in TC and EC eigengene network. **d, g)** The heatmap shows the eigengene adjacencies in TC and EC eigengene networks. Each row and column correspond to an eigengene tagged by consensus module color. Within each heatmap, red represents high adjacency (positive correlation) and blue represents low adjacency (negative correlation) as represented by the color legend. **e)** Bar plot shows the preservation degree of each consensus eigengene as the height of the bar (y-axis) where each colored bar corresponds to the eigengene of the associated consensus module. The high-density value *D* (Preserve TC, FC) = 0.95 indicates the high overall preservation between the TC and EC networks. **f)** Adjacency heatmap of the preservation network between TC and EC consensus eigengene networks, the saturation of the red color indicates correlation preservation of EC and TC module eigengenes.


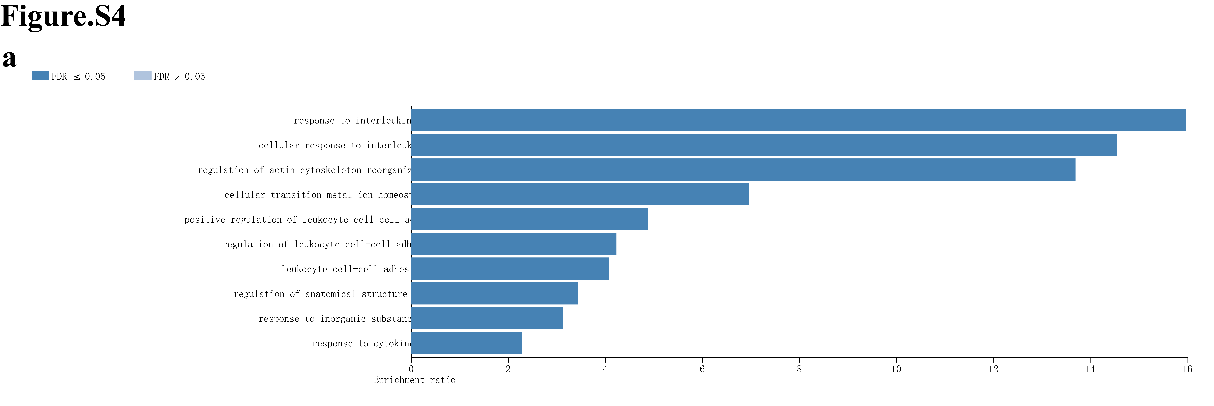


**Figure.S4. The GO enrichment of the overlapping genes. a)** The biological process results.


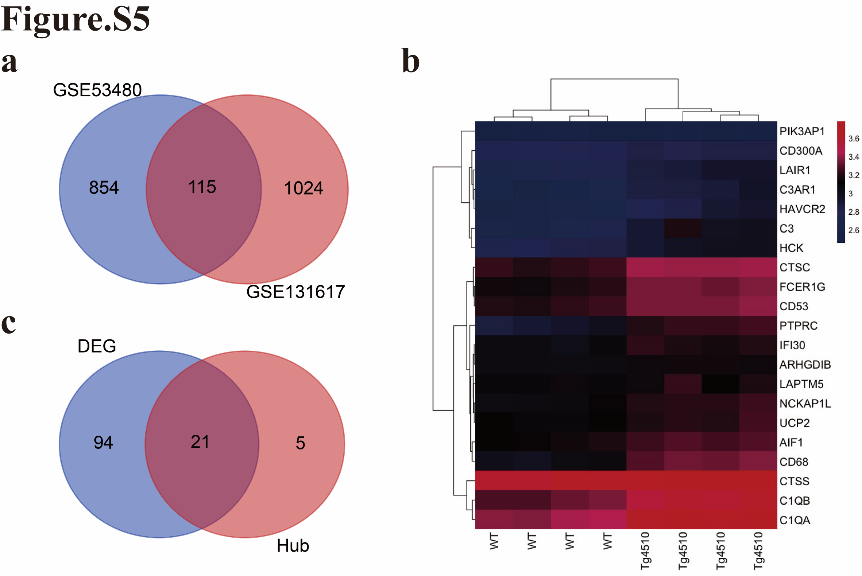


**Figure.S5. The overlapping genes between DEGs of GSE53480 and DEGs of GSE131617. a)** Using veen tools to find the overlapping genes between DEGs of GSE53480 and DEGs of GSE131617. **b)** Heatmap showing the expression of the overlapping genes in a). **c)**Using veen tools to find the overlapping genes between the co-expression genes in two data sets and the 26 DEGs in black module.


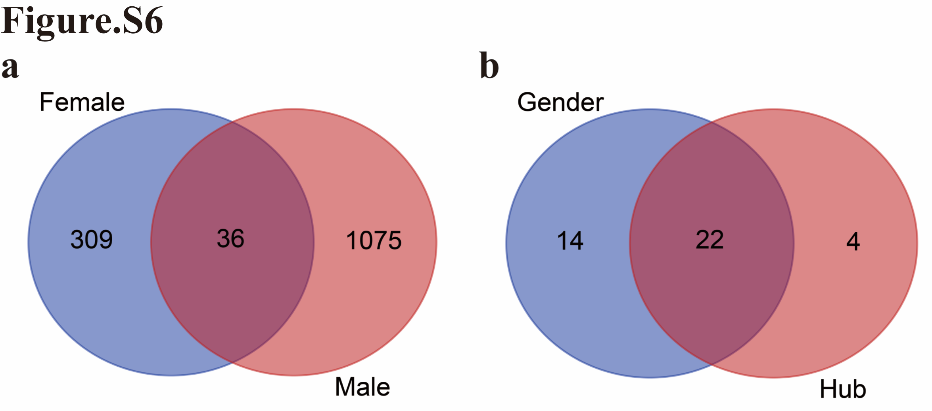


**Figure.S6. The overlapping genes between the genders. a)** Using veen tools to find the overlapping genes between male and female. **b)** Using veen tools to find the overlapping genes between the co-expression genes in genders and the 26 DEGs in black module.


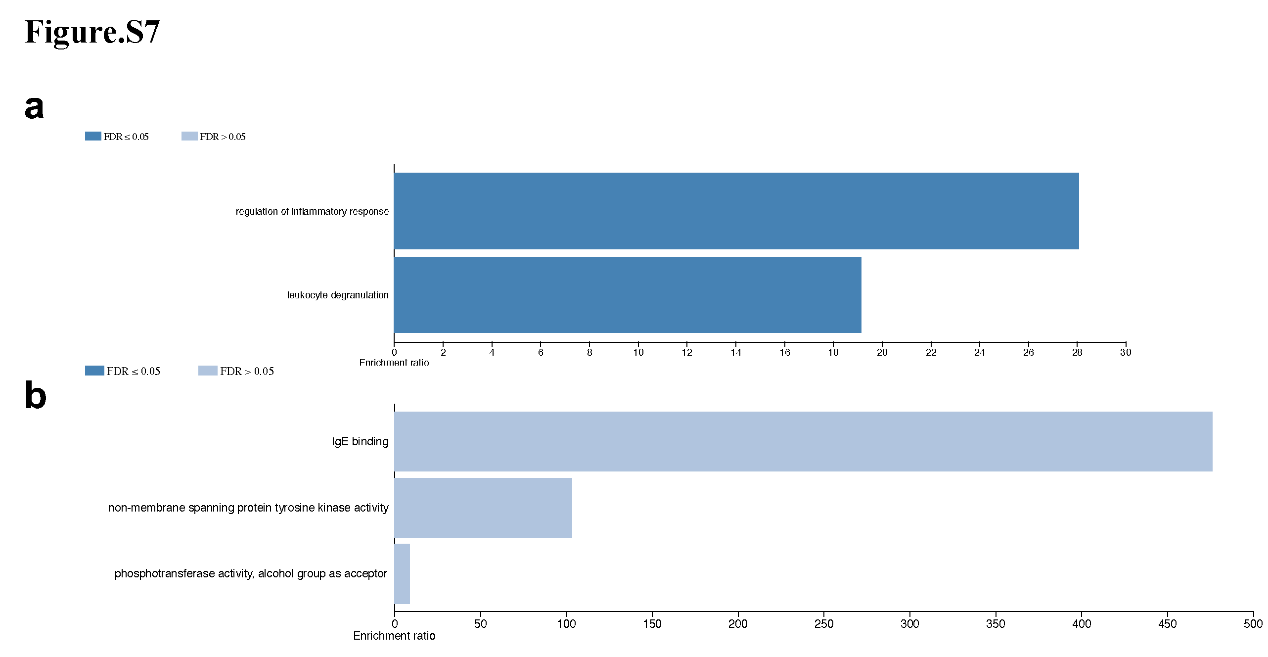


**Figure.S7 The GO enrichment of important genes. a)** The biological process results. **b)** The molecular function results.
